# DTLCx: An Improved ResNet Architecture to Classify Normal and Conventional Pneumonia Cases from COVID-19 Instances with Grad-CAM-Based Superimposed Visualization Utilizing Chest X-ray Images

**DOI:** 10.3390/diagnostics13030551

**Published:** 2023-02-02

**Authors:** Md. Khabir Uddin Ahamed, Md Manowarul Islam, Md. Ashraf Uddin, Arnisha Akhter, Uzzal Kumar Acharjee, Bikash Kumar Paul, Mohammad Ali Moni

**Affiliations:** 1 Department of Computer Science and Engineering, Jagannath University, Dhaka 1100, Bangladesh; 2 School of Information Technology, Geelong, Deakin University, Geelong, VIC 3216, Australia; 3 Department of Information and Communication Technology, Mawlana Bhashani Science and Technology University, Tangail 1902, Bangladesh; 4 Department of Software Engineering, Daffodil International University, Dhaka 1207, Bangladesh; 5 Artificial Intelligence & Data Science, School of Health and Rehabilitation Sciences, Faculty of Health and Behavioural Sciences, The University of Queensland, St. Lucia, QLD 4072, Australia

**Keywords:** coronavirus (COVID-19), respiratory syndrome, convolutional neural network, pneumonia diagnosis, deep learning, healthcare professionals, Grad-CAM

## Abstract

COVID-19 is a severe respiratory contagious disease that has now spread all over the world. COVID-19 has terribly impacted public health, daily lives and the global economy. Although some developed countries have advanced well in detecting and bearing this coronavirus, most developing countries are having difficulty in detecting COVID-19 cases for the mass population. In many countries, there is a scarcity of COVID-19 testing kits and other resources due to the increasing rate of COVID-19 infections. Therefore, this deficit of testing resources and the increasing figure of daily cases encouraged us to improve a deep learning model to aid clinicians, radiologists and provide timely assistance to patients. In this article, an efficient deep learning-based model to detect COVID-19 cases that utilizes a chest X-ray images dataset has been proposed and investigated. The proposed model is developed based on ResNet50V2 architecture. The base architecture of ResNet50V2 is concatenated with six extra layers to make the model more robust and efficient. Finally, a Grad-CAM-based discriminative localization is used to readily interpret the detection of radiological images. Two datasets were gathered from different sources that are publicly available with class labels: normal, confirmed COVID-19, bacterial pneumonia and viral pneumonia cases. Our proposed model obtained a comprehensive accuracy of 99.51% for four-class cases (COVID-19/normal/bacterial pneumonia/viral pneumonia) on Dataset-2, 96.52% for the cases with three classes (normal/ COVID-19/bacterial pneumonia) and 99.13% for the cases with two classes (COVID-19/normal) on Dataset-1. The accuracy level of the proposed model might motivate radiologists to rapidly detect and diagnose COVID-19 cases.

## 1. Introduction

In 2019, the pandemic of COVID-19 emerged as the most contagious disease that causes severe respiratory symptoms infected by SARS COVID-2. This virus was first discovered in Wuhan, which is a province of Hubei, China, on December 31, 2019, and it turned into a pandemic disease soon after [1]. In the first months of 2020, COVID-19 spread across the world through human-to-human transmission. Approximately 4.5 million people died, and the amount of positive confirmed COVID-19 cases across the world was around 218 million to August 2021 according to Worldmeter [2]. The disease generally causes a respiratory infection ranging from mild cold symptoms to severe acute diseases including Severe Acute Respiratory Syndrome (SARS), Middle East Respiratory Syndrome (MERS), and the recently discovered coronavirus (COVID-19) [3]. The symptoms of this disease include fever, dry cough, headache, sore throat, acute pneumonia, organ failure, and Acute Respiratory Distress Syndrome (ARDS) [4].

Generally, special medication and care are not required for many patients infected with COVID-19. The Worldmeter shows that both elderly people and people who already have chronic diseases such as cancer, cardiovascular disease, chronic respiratory diseases and diabetes are more vulnerable to COVID-19 [5]. COVID-19 can also fatally infect people who smoke cigarettes, are exposed to chemical fumes in factories, and suffer from asthma-related complications. The symptoms of this virus are analogous to the common flu in many cases. However, COVID-19 cannot be so easily detected at an early stage. In general, antibiotics are recommended for curing fungal or bacterial infections. However, COVID-19 is unlikely to be medicated by antibiotics due to virus infection. A vaccine or drug that can be 100% effective against the COVID-19 virus is still unavailable. Clinical trials and experiments are still progressing all over the world to discover an effective vaccine.

At present, the most common and reliable technique to diagnose COVID-19 cases is RT-PCR (reverse transcription-polymerase chain reaction). This laboratory method is widely applied to identify COVID-19 instances from respiratory samples, such as oropharyngeal or nasopharyngeal swabs. However, RT-PCR shows low sensibility in the early periods with a prolonged screening period that assists the further transmission of this disease [6]. Furthermore, RT-PCR cost is high, often provides false-negative results, requires more user interaction, and has limited availability.

**Figure 1:**
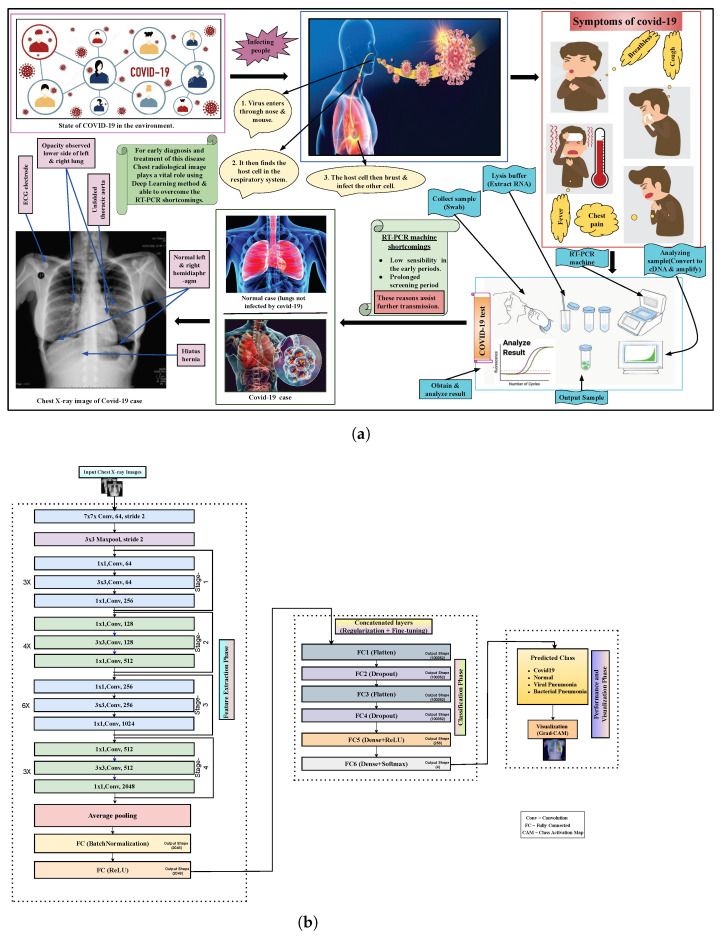
Overview and detection scheme of COVID-19. (**a**) Overview of COVID-19 state in the environment, symptoms, testing, identification with confirmed COVID-19 patient’s chest X-ray sample. (**b**) Modified ResNet50V2 for COVID detection where the 1st part is considered as feature extraction part and then the 2nd part is considered as a classification of different cases images, and lastly, the 3rd part is considered as performance and visualization of the X-ray images part.

Several studies have shown that the deep learning model trained using chest radiography images has produced better accuracy that can possibly overcome the drawbacks of the RT-PCR technique in the near future. To diagnose and medicate this disease, two types of chest radiological images play a vital role: computed tomography (CT) and X-ray [7]. In comparison to X-rays, CT scans need high radiation in the screening process [8]. In this case, although CT scans provide subtle details, X-ray images are available and patients can easily and rapidly have X-ray images in a more cost-saving way. Additionally, an X-ray generates low ionizing radiations and is the less invasive substitute. Therefore, applying X-ray images in order to train deep learning can create a sustainable COVID-19 detection model and alternative to RT-PCR. Considering the benefits of chest X-ray images, we prefer it to CT scans to conduct the investigation of the proposed model. Figure 1a shows the overview of the COVID-19 situation in the environment including symptoms of this disease, testing process, and identification with a confirmed COVID-19 patient’s sample of chest X-ray images.

In this article, we reviewed literature on chest image classifications by conducting various deep learning frameworks based on chest CT scan and X-ray as well as proposed a unique deep learning framework to detect and diagnose COVID-19 cases. The suggested model is capable of differentiating cases with COVID-19 from regular pneumonia and normal images with a higher accuracy level. Developing a deep CNN-based model trained with X-ray images and a Grad-CAM-based visualization approach can aid the radiologist in the early screening of infected patients for quick diagnosis at a low cost. The primary contributions can be summarized as follows:

1. Using a more sophisticated methodology for diagnosis and identifying COVID-19 patients through a deep learning approach, which is based on X-ray images.
2. Developing and examining a deep learning model to automatically detect and early diagnose COVID-19-infected patients in an effective manner.
3. Performing an experimental analysis of the proposed model while classifying COVID-19 cases by using operable X-ray images. It has a lower implementation cost than other existing models that are trained using CT scan.
4. Classifying COVID-19 images as four classes, three classes and binary classes: namely COVID-19, normal, bacterial, and viral pneumonia from normal and regular pneumonia.
5. Demonstrating comparative performance analysis of the proposed work with the other previous state-of-the-art works and Grad-CAM-based visualization marking the most flawless classification results of the COVID-19 cases using the datasets of chest X-ray images.

The rest of this paper is organized as follows: Section 2 provides an extensive overview of the related works. The methodology of the proposed model is demonstrated in Section 3. Experimental performance results with the dataset descriptions are presented in Section 4. A brief discussion of the proposed study is given in Section 5. In Section 6, the conclusion with future directions are provided.

## 2. Related Background

At the beginning of the COVID-19 epidemic, the Chinese clinical centers had experienced inadequate numbers of test kits. Furthermore, the testing kit also generates a high percentage of FN (false negative) outcomes. Therefore, healthcare professionals and researchers were emboldened to build a diagnosis model based on chest CT and clinical results [9]. AI has overcome a long trial period since its emergence, and it has a great ability to identify human activities [10]. The usage of AI in healthcare systems has recently increased a lot, especially in medical imaging technology. Again, medical imaging technology has been widely used in detecting brain tumors [11], cardiovascular disease [12], etc. In addition, it is further being used nowadays for the detection of COVID-19 cases. CT scan and chest X-ray are favorable imaging technology used in medical sectors for image diagnosis. Models based on X-ray imaging can be used for detecting COVID-19 cases in the most densely populated countries such as India, Bangladesh, etc. Researchers suggest performing COVID-19 testing both on the kits and the machine learning model trained with the clinical image to have accurate identification of COVID-19 cases [13,14].

**Figure 2:**
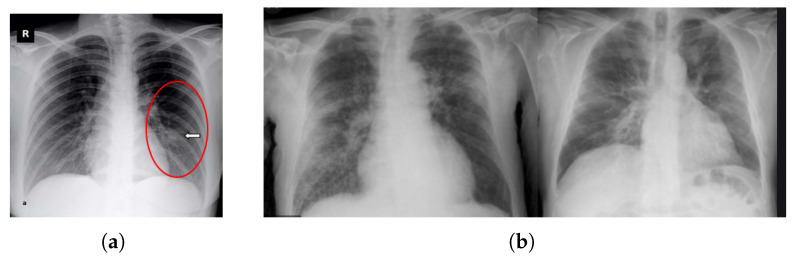
Sample chest X-ray. (**a**) Ground Glass Opacity found (Opacities of infrahilar airspace were found right side in the red circle of COVID-19 patient) [20]. (**b**) Consolidation.

Research has found some variations in X-rays and CT scan images of a patient before the onset of COVID-19 symptoms [15]. Yoon et al. reported that a singular nodule opacity was found in the left side of the lower lung part of one in three patients [16]. Kong et al. [17] reported that on the right side, opacities of infrahilar airspace were found in COVID-19 patients. However, most of the studies showed that mostly GGO (Ground Glass Opacity) is found in COVID-19 patients. Common lung situations combined with GGO are widespread inflammatory and invasive disorders of the lung [18]. Zhao et al. [13] observed that GGO (Ground Glass Opacity) and mixed GGO were found in a maximum number of patients, and they also found vascular dilation and consolidation in the bruised area. Kanne et al. [19] reported that there was multifocal GGO or peripheral focal effects on both lungs of 50–75% of patients. Again, the consolidation of images gives a better view of utilizing the opacity in Figure 2.

Many researchers have already proposed different deep learning models trained with radio images to identify COVID-19 cases because of the availability of radiography images. In addition, big data and IOT (the Internet of Things) technology are being used for the prediction and detection of various current issues [21]. A deep learning framework for IoT-based early COVID-19 assessment was proposed [22], which combined IOT and two CNN (Faster R-CNN and ResNet-101) networks and achieved a detection accuracy of 98%. Awam et al. proposed [23] a deep learning approach with a big data-enabled model to detect COVID-19 from chest X-ray images. This study deployed a Deep Transfer Learning (DTL) technique to COVID-19 chest X-ray images using the Apache Spark system as a large-scale data platform and three convolutional neural network (CNN) architectures—InceptionV3, ResNet50, and VGG19. Sohail et al. proposed [24] transfer learning for the Rising Rigorousness Scores using COVID-19 variants. They demonstrated using transfer learning that the cardiovascular fatality rates and the stringency index were the most significant and appropriate variables to create the network for the forecasting of the COVID-19 mortality rates. Deep learning procedures have already been applied in many areas including skin cancer classification [25], brain disorder classification [26], classification of pneumonia using X-ray images of the chest [27,28], and lung and colon cancer [29,30]. An architecture based on the CNN method [31] is widely used in detecting COVID-19 cases. Ghavami et al. [32] proposed an interpretable artificial intelligence method using chest CT in order to detect COVID-19 where they considered three classes: COVID-19 patients, healthy patients, and non-COVID lung infections. A hybrid deep transfer learning model was proposed [33] using fifteen pre-trained convolutional neural networks (CNNs)-based architectures with chest CT scan images. Some studies [34,35] also worked on CT scan images to detect COVID-19 cases. Some studies [36,37,38,39,40,41,42] worked on both chest CT scan and X-ray cases to detect and diagnose COVID-19 combined. Mukherjee et al. [43] proposed a CNN-based tailored deep neural network (DNN) using CT scan and X-ray images with comprehensive accuracy with 96.13% for X-ray and 95.83% for CT scan. An ideal fine-tuned VGG-19 model utilizing chest CT and X-ray was presented for detecting and diagnosing COVID-19 [44].

Focusing deeply on the study based on deep learning with chest X-ray, some recent works have also been included in this research. Deep learning methods are used [45] to automatically identify COVID-19 from digital chest X-ray images. Mousavi Z et al. [46] proposed a technique that divides two to four classes into seven separate scenarios using chest X-ray images, including bacterial, viral, healthy, and COVID-19 classes. Luz et al. [47] proposed an efficient deep learning model to detect COVID-19 from X-ray images. The primary objective of this work is to formulate a method for the issue of COVID-19 screening in chest X-rays that is accurate yet effective in terms of memory and processing time. Al-Waisy et al. [48] suggested a hybrid framework (COVID-CheXNet) based on deep learning for classifying COVID-19 cases using chest X-ray images. Chen et al. [49] worked on a deep transfer learning model named VGG-16 for detecting COVID-19 in X-ray images by considering two classes including COVID-19 and non-COVID-19. Wang et al. [50] suggested a model (COVID-Net) to identify COVID-19 cases from pneumonia and normal cases by using an unbalanced dataset with an accuracy of 93.3%. An integrated stacked model named InstaCovNet-19 was proposed by Gupta et al. [51]. They used various transfer learning models including InceptionV3, Nasnet, Xception, MobilenetV2, Resnet101 and then integrated all these models into a stack-like architecture. Moreover, for the classification of images, they used three different classes and achieved a pretty higher accuracy of 99.08% for three classes and 99.53% for two classes. Ammar et al. [52] presented six conventional transfer learning models including MobileNetV2, DenseNet12, Xception, ResNet50V2, VGG16 and ResNet152V2 by using three classes. They compared their six models and achieved the highest accuracy of 91.28% for MobileNetV2. An unbalanced dataset of three classes with 1345 normal images, 3632 pneumonia images and 490 COVID-19 images was used by Jain et al. [53]. They compared three deep learning models including ResNeXt, Xception net, and Inception net V3 and obtained the highest accuracy of 97.97% for the Xception model. Mohammadi et al. [54] used some pre-trained transfer learning models (VGG-16, MobileNets, VGG-19 and InceptionResNetV2) for detecting COVID-19 cases and among the models, MobileNets performed well for two classes with COVID-19 images and normal images. An AlexNet with the combination of SVM architecture was presented in [55] where a fine-tuning process on the suggested architecture was performed to identify COVID-19 cases from pneumonia and normal cases. Ouchicha et al. [56] proposed a deep CNN model named CVDNet to identify COVID-19 infection from pneumonia and normal images. They used a five-fold cross-validation process to validate their experiment. An automated detection technique (DarkCovidNet) was presented by Ozturk et al. [20] that was employed on two-class classification (COVID-19 vs. normal cases) and multi-class categories (COVID-19 with normal and pneumonia cases) acheived outstanding accuracy of 98.08% in binary class. However, any preprocessing operation on X-ray image data such as augmentation, etc. was not performed in this study. A deep CNN model named CoroNet was introduced by Khan et al. [57] to identify and categorize COVID-19 cases. A conventional model named Xception was developed by them to strengthen their experiment. They used X-ray images for their experiments with four categories including confirmed-COVID-19, normal and regular pneumonia (caused by virus and bacteria). An architecture of ResNet50 with SVM was proposed in [58] where they used a small dataset of COVID-19 negative cases and positive cases with 96.38% accuracy. An overview of the previous studies is presented in Table 1.

After reviewing the above existing work, it is evident that most of the experiments were trained with an unbalanced dataset as well as an inadequate dataset of the cases with COVID-19. These things may lead to a CNN-based architecture that has overfitting issues, and it does not disclose the real classification performance of the suggested approaches due to the small dataset. Moreover, some of the works trained raw images directly to their models with no preprocessing, augmentation was performed on raw data, and thus generalization error of the network was increased as well as training benefit was decreased. Furthermore, most of the experiments were conducted using conventional pre-trained deep learning models and trained their model using two or three classes. So, in these cases, we addressed the drawbacks by creating a balanced dataset and instead of using raw images, preprocessing and augmentation were completed on our collected images. Therefore, this encouraged us to develop a conventional deep learning model by effective fine-tuning with optimizing hyper-parameters to make the model more robust. In addition, multi-class comparisons such as four-class, three-class and two-class comparisons as well as Grad-CAM visualization among the image classes would make our experiments more effective.

## 3. Methodology

**Figure 3:**
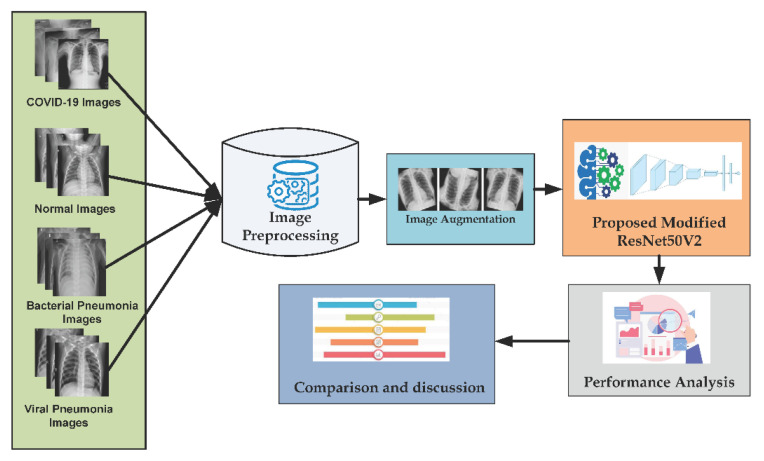
Overview of the proposed research methodology.

This section includes the description of preprocessing, augmentation of our dataset that is fed to the model to classify COVID-19 cases, the CNN model, and finally, the architecture is designed prior to carry out experiments to analyze the performance. The overview of our suggested methodology is exhibited in Figure 3. In this illustration, first of all, the collected dataset of different class images of cases with COVID-19, regular pneumonia, and normal cases used as input samples. After that, preprocessing of this collected image is performed based on resizing criteria. Then, augmentation on this dataset is completed to increase the diversity of the image data based on some criteria such as rescaling, zooming, shearing and flipping. Afterward, by extending and fine-tuning the ResNet50V2 CNN architecture, the study’s proposed model is developed. ResNet50V2 [65] is an enhanced version of ResNet50 [66] that outperforms ResNet50 and ResNet101 [67] on the ImageNet datasets. A modification or adjustment was applied to ResNet50V2’s extended formulation of the links within blocks. In terms of feature extraction, deeper models do well. However, on account of the characteristics of feed-forward (inputs are passed to the model to gain prediction outcome from complex computations in the model) and back-propagation (weight of the parameters’ update on the basis of prediction result), which is the substance of experimenting deep learning models, highly deep models are difficult to train on account of exploding or vanishing gradients. In this case, ResNets solve this drawback by formulating a residual connection, decreasing the impact of exploding or vanishing gradient and thus enabling the highly deep models to perform better. ResNet50V2 has taken out the last non-linearity, consequently, making the way of input to output such as identity connection. As a result of selecting and developing the ResNet50V2 architecture with extended layers, fine-tuning, and regularization, the proposed model becomes more robust and feasible when compared to other well-studied RESNETS models presented in Table 1. Based on the proposed model, the dataset is classified into binary, three and four-class categories. Then, the performance analysis of the model is completed with different perspectives. Finally, based on the performance results, a general comparison between used datasets is made as well as exploration of the best dataset is also identified.

### 3.1. Data Preprocessing

The acquired data’s (COVID-19 images) original size had 1024*1024 pixels, and the other images of 3 classes had diverse pixels for the images. ImageNet for pre-trained models will have inputs that are less than or even to 224*224. In case of deep transfer learning models, the inputs should be adopted to pre-trained models. In this manner, for rigorous inquisition purpose, all images were resized to 224*224 pixels to assist the training model to run faster.

### 3.2. Data Augmentation

Augmentation of data is a process that allows practitioners to notably improve the diversity of the data (e.g., images) for training models instead of collecting the new data. Image augmentation techniques may decrease the network generalization error and increase the training amenities as well as deal with the data overfitting issues. In this research, augmentation procedures [68] on image data were completed for the enhancement of the images based on rescale, horizontal flip, zoom and shear operations. These techniques were completed by using the functionality of ImageDataGenerator from TensorFlow, Keras framework [69,70]. In augmentation settings, we set the value of the above given criteria following rescale = 1/255, shear range = 0.2, zoom range = 0.2 and horizontal flip = True have been considered.

### 3.3. Transfer Learning with Convolutional Neural Networks

The working methods of the suggested model have used a deep transfer learning framework as a base. Recently, transfer learning-based CNN models have gained popularity among researchers to solve various computer vision problems. These models are widely used in medical diseases, including diagnostic [71], industries, and agriculture over the last decades [72,73]. In this article, a CNN-based deep transfer learning model is developed and used for X-ray image classification.

#### 3.3.1. Convolutional Layer

The main building block of the CNN (convolution neural network) is the convolution layer. It conducts convolution operation (denoted by *) in place of simple matrix multiplication. Its parameters are built up with a set of learnable filters, and these filters are also called kernels. The major task of this layer is to identify features that are found in the native regions of the input samples (e.g., image) and generate a feature map that reduces the appearance of the detected features in the input samples. The typical convolution operation is expressed according to Equation (1).

(1) $$F\left(i,j\right)=\left(I*K\right)\left(i,j\right)=\sum_{m}\sum_{n}I\left(i+m,j+n\right)K\left(m,n\right)$$

The outcome of each layer of convolution is compiled using a function named the activation function for the establishment of non-linearity. ReLU (rectified linear unit) generally calculates the activation function by thresholding the input to zero. It can also be said that if the input is less than 0, ReLU gives 0 output and otherwise gives the raw output. It can be represented mathematically according to Equation (2).

(2) f(x)=max(0,x)

So, if the input value of *x* is less than zero, the function *f*(*x*) generates output 0, and if the input value of *x* is greater than or equal to zero, then the function *f*(*x*) generates output 1.

#### 3.3.2. Pooling Layer

In convolutional neural network (CNN), pooling layers are the significant portion of convolution layer sequence. These layers minimize the spatial dimensions of the input samples by assembling the outputs of the neuron bunches at one layer and turning them into a single neuron in the next layer. The operation of pooling layers involves sliding a 2D (dimensional) filter over every channel of the feature map and summarizing the features placed within the field covered by the filter. There are some various pooling layers that are used in convolutional neural networks: namely, max pooling, global pooling layers, L2-norm pooling, and average pooling. Max pooling is the most general pooling technique compared to others that generates maximum value while it is used in the input zone.

#### 3.3.3. Fully Connected Layer

A fully connected layer is an indispensable component of a convolutional neural network (CNN) where every neuron from the preceding layer is connected to every neuron in the subsequent layer and imparts to the prediction of how closely each value matches with each particular class. Then, the output of the last FC (fully connected) layer is linked to a function called the “activation function” that generates output class scores. Various classifiers are used in CNN such as Sigmoid, SoftMax, SVM (support vector machine), etc. The probability ordination of n number of output classes can be calculated by the SoftMax function shown in Equation (4).

(3) $$Z^{k}=\frac{e^{x^{k}}}{\sum_{i=1}^{n}e^{x^{n}}}$$

The given notation Table 2 explains all the used math symbols for better understanding.

### 3.4. Proposed Architecture with Six Extra Layers

The base model in this study consists of four major stages with some additional stages. There are three layers in the first stage of convolution: a 1*1,64 kernel, a 3*3,64 kernel, and finally a 1*1,256 kernel. These three layers are repeated a total of three times, giving us nine layers in this step. Following that, we see a kernel of 1*1,128 followed by a kernel of 3*3,128 and finally a kernel of 1*1,512. This procedure was performed four times for a total of 12 layers. After that, there is a 1*1,256 kernel, followed by two more kernels with 3*3,256 and 1*1,1024; this is repeated six times, giving us a total of 18 layers. Thereafter, a 1*1,512 kernel was added, which was followed by two more kernels of 3*3,512 and 1*1,2048. This process was performed three times, giving us a total of nine layers.

The Residual Blocks or stages idea was used by in this study to address the issue of the vanishing/exploding gradient. In this network, a procedure is employed called skip connections. The skip connection skips some intermediate layers, allowing activations on one layer to relate to activations on succeeding ones. An extra block is produced as a result. In order to build resnets, these extra blocks are piled. The advantage of using this type of skip link is that regularization will bypass any layer that reduces the performance of the architecture. Consequently, vanishing or growing gradient problems are not encountered during training of an exceedingly deep neural network. The stages of this ResNet architecture reduce the complexity and address the degradation while maintaining good performance.

However, we tuned and improved the base architecture of ResNet50V2 to make our proposed framework more robust and effective. In that case, two dropouts, two flattened layers, and two fully connected (marked as fc) dense layers are assembled at the bottom as shown in Figure 1b. First of all, the original ResNet50V2 top layer was modified. to specify the images’ specific input. Secondly, the pre-trained ResNet50V2 network is concatenated with several layers. The additional layers were then treated to regularization as well as fine-tuning techniques. The additional layers make the overall model more feasible and robust. The flattened layer converts the previous layer data into a 1D (one dimensional) array and makes the array data for the input of the next layer. We flatten the output of the previous convolutional layer to generate a single-length feature vector. The next adding layer we used is the dropout layer. This layer works as a mask that avoids the contribution of several neurons toward the subsequent layer and leaves unconverted all others. We used the dropout layer because it is so essential in training the deep CNNs model by controlling the overfitting issues. After that, two dense layers are added to optimize and generate the output. In terms of the dense layer, each neuron receives input from all of the neurons that exist in the preceding layer—in this way, they are densely connected. The dense layer is also called the fully connected layer. Moreover, the direct usage of hyper-parameters in the model becomes critical because they directly control the nature of the model.

So, in this case, we fine-tuned the hyper-parameters in order to make our model perform well. The proposed model uses an image size of (224 × 224) and the weights of the model are ImageNet with Adam optimizer [74], the learning rate of 1 × 10^−5^, the batch size of 32, and the number of epochs used for each of the experiments is 50. In addition, SoftMax (activation function) [75] is used in this study to classify the images not only in two-class but also multi-class. The summary of the proposed model is presented in Table 3.

## 4. Experimental Results

### 4.1. Dataset Description

**Figure 4:**
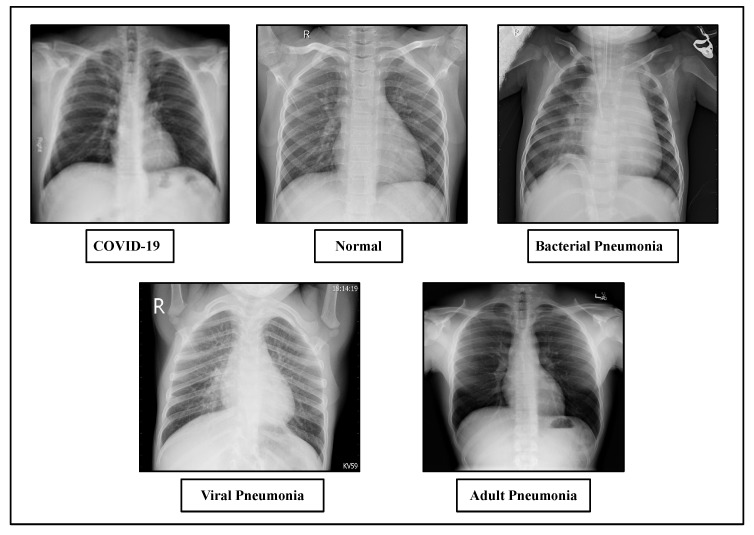
Sample chest X-ray of COVID-19, normal, viral pneumonia, bacterial pneumonia and adult pneumonia cases.

For our experiment, the dataset has been created from three different publicly available sources. The proposed dataset consists of a total number of 4593 chest X-ray images. To avoid the class imbalance problem, we have used a balanced dataset. First of all, the dataset includes 1143 chest X-ray images of COVID-19 patients collected from Kaggle’s repository “COVID-19 Radiography Database” provided by Tawsifur Rahman [76] and 1150 normal, 1150 bacterial pneumonia and 1150 viral pneumonia chest X-ray images from “Chest X-Ray Images (Pneumonia)” provided by Paul Mooney [77]. We have also collected some adult pneumonia dataset from kaggle’s repository “RSNA Pneumonia Detection Challenge” provided by the Radiological Society of North America [78]. We have used three datasets for our experiment: namely, Dataset-1, Dataset-2 and Dataset-3. Table 4 displays the Dataset-1 distribution for each subset with training and testing the data. In Dataset-1, for training purposes, we have used 60% dataset samples for training purposes and the remaining 40% samples are used for testing purposes. On the other hand, Table 5 shows the Dataset-2 distribution where 100% of the dataset samples are used for training and 40% of the data split from the training dataset is used for testing purposes. Figure 4 displays sample images of chest X-rays with different cases.

### 4.2. Experimental Configuration and Implementation

The proposed model is implemented in Keras with Tensor flow [70] GPU support. Total experiment, training, and testing are performed on Google Colaboratory environment with some supporting equipment including a Tesla T4 graphics card, 12.72 GB RAM, and 66.40 GB Disk space.

The gradient descent optimization technique Adam Optimizer [74] was employed in the suggested architecture. When it comes to solving issues requiring a large number of data samples or parameters, this strategy is highly effective. Adam differs from the standard stochastic gradient descent. The stochastic gradient descent algorithm employed a constant learning rate (called alpha) for all weight changes. On the other hand, Adam used the advantages of AdaGrad and the RMSProp algorithm during training to repeatedly change network weights. A few of the setting options for Adam are alpha, beta1, beta2, and epsilon. In this case, alpha was interpreted as the learning rate or step size. Consequently, the suggested model made use of an adjustable learning rate using a 1 × 10${}^{-5}$ Adam Optimizer. We have implemented our model to classify the cases of COVID-19 from other cases (bacterial pneumonia, normal and viral pneumonia). We have used different types of class-wise classification categories including four-class, three-class and two-class categories to classify COVID-19 instances. In four-class categories, the classification has been made on COVID-19 cases vs. others (normal cases, pneumonia with bacterial cases and pneumonia with viral cases). In three-class categories, the classification has been made on COVID-19 cases, normal cases and bacterial pneumonia cases, and another fold includes COVID-19 cases, normal cases and pneumonia with viral cases. Again, in two-class categories, the classification has been made in three folds including COVID-19 with normal cases, COVID-19 with bacterial pneumonia cases and COVID-19 with viral pneumonia cases. Again, the four-class classification has been performed on both Dataset-1 and Dataset-2.

### 4.3. Evaluation Metrics

In this study, four performance metrics are conducted to evaluate the performance results of the proposed architecture. The used evaluation measures are accuracy, precision, sensitivity (recall) and f1-score. Hence, the mathematical equations of the metrics are exhibited below in the following Equations (4)–(7), respectively.

(4) $$Accuracy=\frac{\left(TP+TN\right)}{\left(TP+FP+FN+TN\right)}$$

(5) $$Precision=\frac{TP}{\left(TP+FP\right)}$$

(6) $$Recall=\frac{TP}{\left(TP+FN\right)}$$

(7) $$F1-Score=\frac{\left(2*Precision*Recall\right)}{\left(Precision+Recall\right)}$$

where *TN*, *TP*, *FN*, *FP* and represent true-negative, true-positive, false-negative and false-positive, respectively.

### 4.4. Normalization of Confusion Matrix

Normalization of the confusion matrix is also performed in this research. Generally, the term “normalized” indicates that each class or group is represented by having 1.00 sample. In this process, the precision values are calculated by considering the column sum of each value or sample assigned to a certain class, and then, the diagonal values are divided by these sums. Again, the diagonal values of the matrix are represented as recall or sensitivity values. These recall values can be achieved considering the rows sum of each value or sample assigned to a certain class, and then, the diagonal values are divided by these sums, respectively.

### 4.5. Classification Performance on Dataset-1

**Figure 5:**
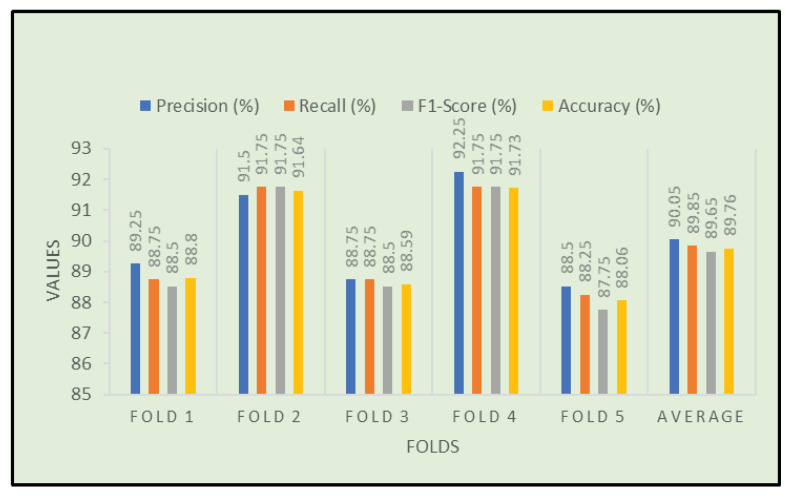
Performance results of the suggested model on each fold.

**Figure 6:**
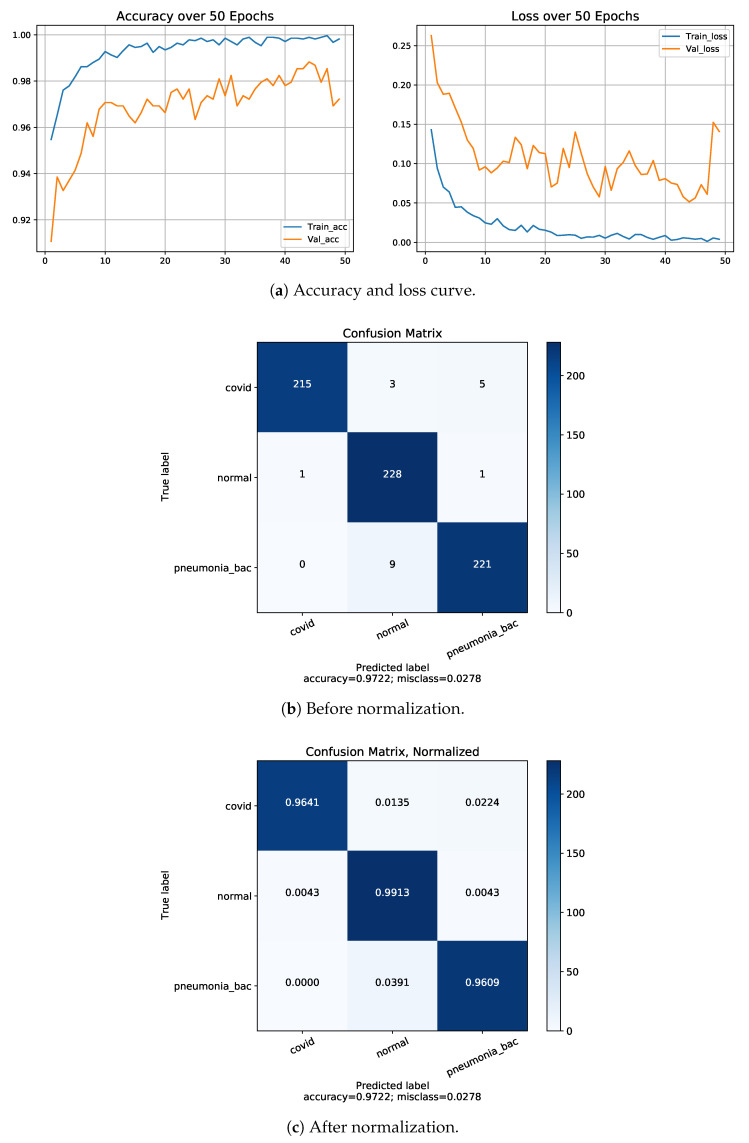
Results of Dataset-1 for 3-class: COVID-19/Normal/bacterial pneumonia.

**Figure 7:**
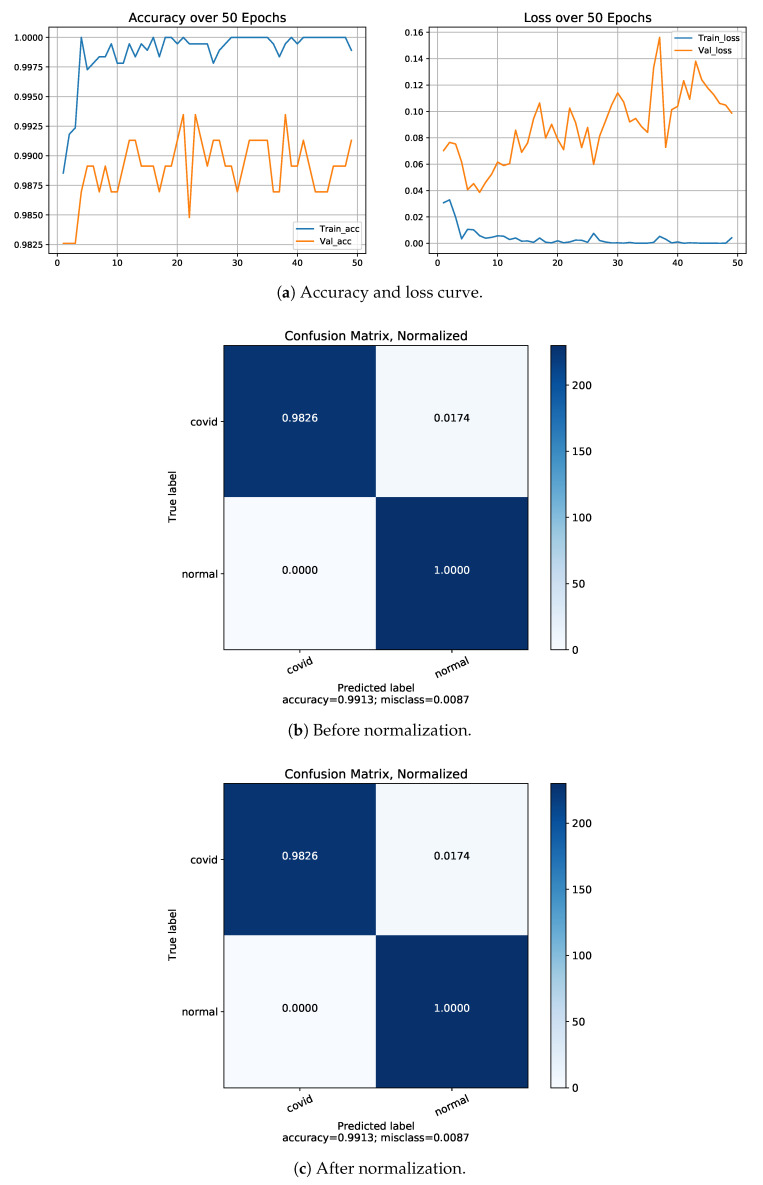
Results of Dataset-1 for 2-class: COVID-19/normal.

On the basis of the given above performance metrics, our proposed model has shown 88.79% accuracy for four-class classifications including COVID-19, normal cases and regular pneumonia (bacterial and viral) cases, 97.22% and 93.11% accuracy for three classes (COVID-19/pneumonia with bacteria/normal) and another with (COVID-19/pneumonia with viral/normal), 99.13%, 98.47% and 98.91% accuracy for two classes firstly, COVID-19/normal, secondly, bacterial pneumonia /COVID-19 and thirdly, viral pneumonia cases / COVID-19 using Dataset-1. From above all the comparison, we have obtained the lowest accuracy on four classes which is 88.79% and the highest accuracy on two classes (COVID-19/normal), which is 99.13%. Although in four classes, classification accuracy is low, but the precision, sensitivity (recall) and f1-score are comprehensively high. The class-wise evaluation of the proposed model is demonstrated in Table 6. Figure 5 represents the graphical representation of the proposed model on each fold by applying 5-fold cross validation approach. The accuracy, loss curve and confusion matrix of three-class classification is two-fold (one consists of (COVID-19, pneumonia with viral cases and normal cases), and another fold consists of (COVID-19, pneumonia with bacterial and normal cases)), while the two-class with three folds includes COVID-19 with normal cases, COVID-19 with viral pneumonia cases and COVID-19 with bacterial pneumonia cases classifications; these are presented in Figure 6 and Figure 7, respectively. A comparison of performances of the proposed model trained with both Dataset-1 and Dataset-2 having four classes is illustrated in Table 7. For the rest of the folds of different classes’ training and validation accuracy, the loss results are presented in Table 8.

### 4.6. Classification Performance on Dataset-2

**Figure 8:**
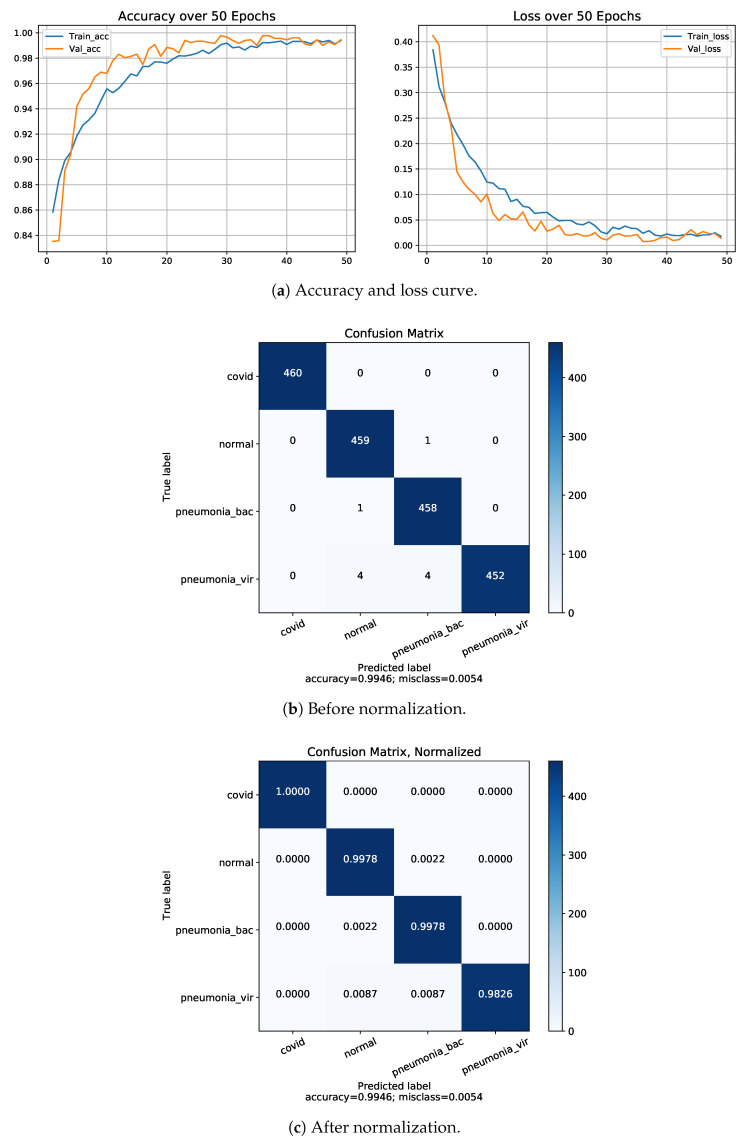
Results of Dataset-2 for 4-class COVID-19/normal/bacterial pneumonia/viral pneumonia.

We have fed our second dataset to the proposed model. The model trained with Dataset-2 has shown better accuracy, which is around 99.46%. The performance metrics on Dataset-2 have been shown to be relatively high compared to Dataset-1 having the same number of classes. The corresponding accuracy, loss curve and confusion matrix of four classes using Dataset-2 is presented in Figure 8.

### 4.7. K-Fold Cross Validation

**Figure 9:**
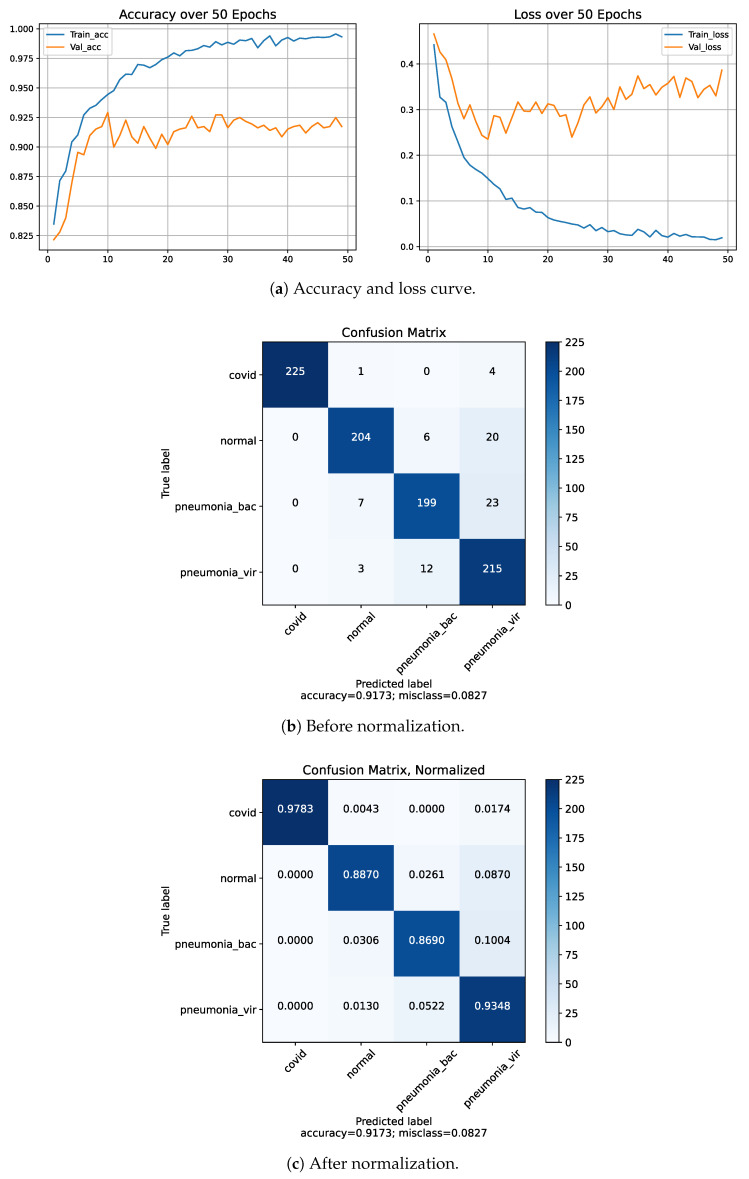
Results of Dataset-1, fold-4 for 4-class COVID-19/normal/bacterial pneumonia/viral pneumonia.

We have applied a five-fold cross-validation approach on a four-class classification problem. In that case, we used 80% data for our training purpose and 20% data for validation. The procedures are repeated 5 times. The final performance is achieved by taking the average of each fold’s values as shown in Table 9. The classification results of fold-4 using four classes are shown in Figure 9. After applying the 5-fold cross-validation procedure, we achieved the highest accuracy of 91.73% on fold-4 and the lowest accuracy of 88.06% on fold-5 among the five folds. Moreover, the overall average accuracy we achieved was 89.76% alongside 90.05% precision, 89.85% recall and 89.65% F1-score, respectively.

### 4.8. Classification Performance on Dataset-3

**Figure 10:**
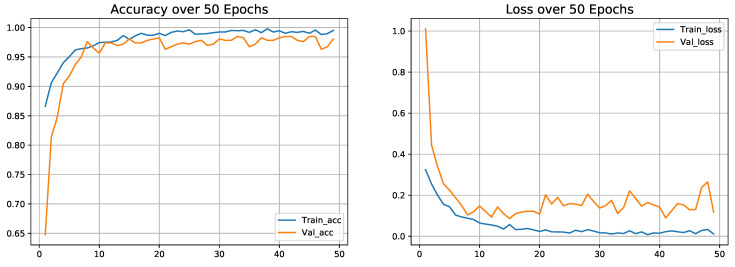
Accuracy and loss curve results on fold-5 of Dataset-3.

**Figure 11:**
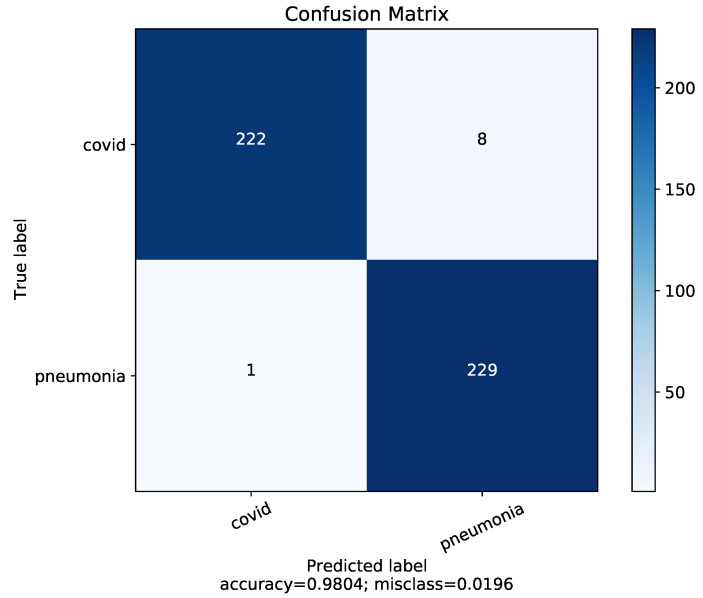
Confusion matrix on fold-5 of Dataset-3.

Dataset-3 consisted of two class where 1143 COVID-19 and 1150 adult pneumonia chest X-ray images are used for the experiment to distinguish between COVID-19 and adult pneumonia cases. We validated the experiment (COVID-19 vs. adult pneumonia) by using k-fold validation where 80% of the image samples are used for training purposes and the remaining 20% are used for testing purposes. The experiment results are shown in the Table 10. From Table 10, the proposed model achieved an average of 98.26% accuracy when differentiating COVID-19 vs. adult pneumonia cases with average precision, recall and F1-score of 98.4%, 98.4% and 98.3%, respectively. The accuracy and loss curve with the 5-fold confusion matrix is illustrated in Figure 10 and Figure 11.

## 5. Discussion

In this study, we have proposed a deep transfer learning model based on ResNet50V2 architecture for detecting COVID-19-affected cases from other cases using chest X-ray images. We have used two datasets for conducting experiments of the model. The first dataset was used to classify images based on four-class cases, three-class cases and binary-class case categories, respectively. On the other hand, the second dataset was applied only on four-class categories to check the model’s robustness. Our proposed model obtained 88.79% accuracy in four classes including COVID-19, normal cases and regular pneumonia (bacterial and viral) cases, 97.22% and 93.11% accuracy in three-class cases (COVID-19 with normal/bacterial pneumonia cases and COVID-19 with normal case/viral pneumonia), and 99.13%, 98.47% and 98.91% accuracy for two classes (COVID-19/normal cases, COVID-19/bacterial pneumonia cases and COVID-19/viral pneumonia) using Dataset-1. Furthermore, a high accuracy of 99.46% with high precision, sensitivity and F1-score values of 99.5%, 99.5% and 99.5% were achieved, respectively, with four-class classification using Dataset-2. On Dataset-3, we achieved an average of 98.26% accuracy when differentiating COVID-19 vs. adult pneumonia cases with average precision, recall and F1-score of 98.4%, 98.4% and 98.3%, respectively. As the recall value of the proposed system is high, it indicates that the lower amount of FN (false negative) and the lower number of false-negative cases foster the experimental results. Table 11 shows the comparisons results between the suggested model and other traditional pre-trained models where the proposed model (on Dataset-1) has achieved better precision, recall and F1-score on the four, three and two-class categories, respectively. Again, we only evaluate the proposed model (on Dataset-2) using the four-class category and we achieve comprehensively higher accuracy than the other pre-trained models and proposed model (on Dataset-1). We find that we obtain relatively lower accuracy for the four-class category compared to three and four-class on Dataset-1; hence, the model will deliver good accuracy for three- and two-class accordingly, as it gives higher accuracy for the four-class category on Dataset-2. As a result, when we ran our test on Dataset-2, we did not take into account the dataset for the three and two-class categories.

The experimental results obtained by our proposed architecture are higher than previous state-of-the-art procedures in this literature. Comparisons among the proposed model and the other preceding studies are shown in Table 12. A multi-dilation CNN model named CovXNet was presented by Mahmud et al. [64] in detecting and categorizing COVID-19 instances from other cases. This model acquired an accuracy 90.2% for four classes considering COVID-19 cases, normal cases, bacterial pneumonia cases and viral pneumonia cases, and it acquired 97.4%, 94.7%, and 87.3% for two different classes including normal vs. COVID-19, bacterial pneumonia with COVID-19 and COVID-19 with viral pneumonia cases. A model CoroNet was suggested by Khan et al. [57] for COVID-19 detection which demonstrated 89.6% accuracy for four-class categories including COVID-19 with normal cases as well as bacterial and viral pneumonia cases and 99% accuracy in binary class (COVID-19 and normal). The above studies did not deal with more patient data as well, as they used an unbalanced dataset, whereas we used more patient images with the balanced dataset for our experiment and obtained respective accuracy compared to these studies as presented in Table 11 and Table 12.

Arsenovic et al. [60] presented a model named ResNetCOVID-19 which obtained 94.1% accuracy in three-class cases (COVID-19, bacterial pneumonia cases and normal). They did not consider data consisting of viral pneumonia whereas our model not only considers the data containing pneumonia with viral cases but also compares the data among the other categories as well as achieved better accuracy compared to those on the same classes, as shown in Table 9. A comparative analysis of various deep learning models was experimented by Ammar et al. [52] using six pre-trained models with 91.28% accuracy on MobileNetV2 for three-class classifications. Instead of using the conventional pre-trained model, we used the tuned ResNet50V2 model and achieved better results as presented in Table 9. An automated COVID-19 detection system named DarkCovidNet was proposed by Ozturk et al. [20] which acquired 98.08% accuracy for the binary class and 87.02% accuracy for three classes. They did not apply any augmentation techniques in their raw data. As the augmentation on data increases the robustness of deep learning models, by applying the data augmentation technique, we obtained better accuracy compared to them.

A COVIDX-Net architecture was suggested by Hemdan et al. [79] for the identification of COVID-19 cases from the non-COVID-19 cases, and they obtained the highest accuracy of 90% for both VGG19 and DenseNet201. The above studies used conventional pre-trained networks or models to classify COVID-19 and non-COVID cases, whereas we modified a pre-trained ResNet50V2 model and achieved comprehensive accuracy on two-class categories, as shown in Table 11. Sethy et al. [58] proposed a model which combined a deep transfer learning model ResNet50 and SVM to identify certain COVID-19 from negative COVID-19 cases with an accuracy of 95.38%. They used an ancient transfer learning model, and for the classification of cases, they used SVM, whereas in our research, we used an upgraded version of ResNet50 and modified it for better performance. Moreover, instead of using the SVM classifier, we used SoftMax and achieved higher accuracy.

### Discriminative Visualization by Proposed Model Using Grad-Cam

**Figure 12:**
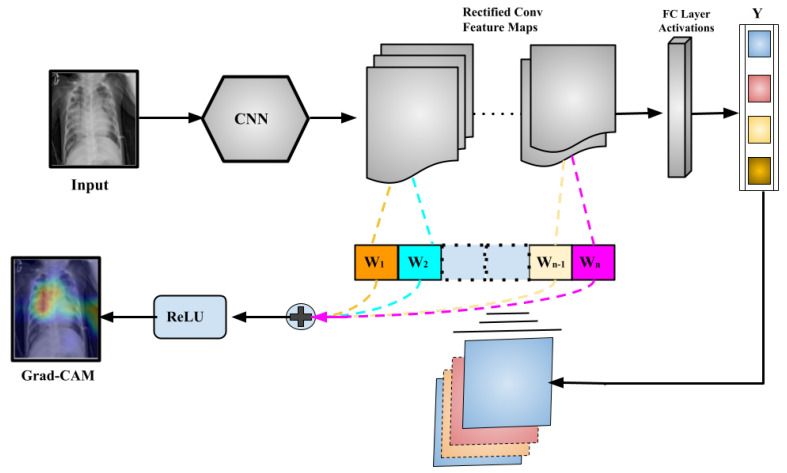
A general illustrative diagram of Gradient Weighted Class Activation Mapping (Grad-CAM).

The proposed model is combined with the gradient-based class activation mapping (Grad-CAM) approach [80] to construct the class activation mapping to localize the specific region of the X-rays that mostly prompted the decision, as illustrated in Figure 12.

**Figure 13:**
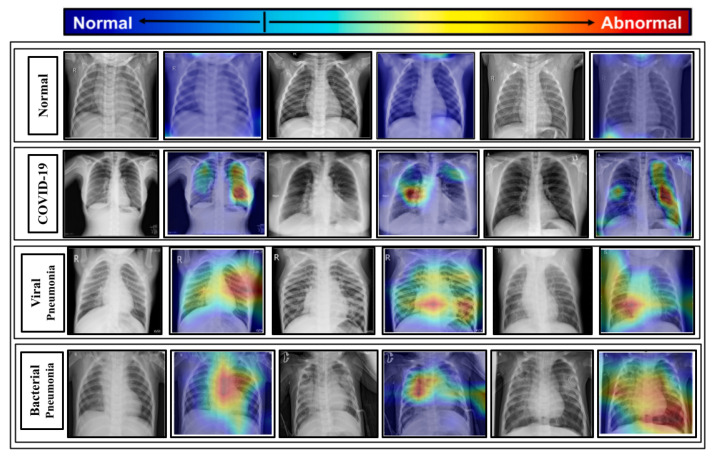
Significant portions of the test chest X-rays that induce the decision are localized by imposing the class activation heatmap obtained from the proposed model.

Such localizations are further investigated by superimposing the heatmap with the input X-rays to evaluate the network’s learning from a clinical standpoint. Some of the chest X-rays with the imposed localization are displayed in Figure 13.

The following are some of the key findings:

- By considering the normal X-rays, there is no opacity in normal X-rays, which distinguishes normal cases from all types of pneumonia cases who have opacities with various types [81,82,83]. For normal X-rays, no substantial region is localized, as shown in Figure 13. Since it is more recognizable, it is easier to distinguish from the other patients.
- When looking at the heatmaps for classical viral pneumonia, it is observed that our proposed model has localized the regions with bilateral multifocal Ground Glass Opacities (GGO) as well as patchy consolidations in some cases. These localized characteristics are also commonly recognized as radiological features of classic viral pneumonia [9,84,85].
- Localized activation heatmaps in the event of bacterial pneumonia generally involve opacities with consolidation on the lower and upper lobes. Furthermore, both unilateral and bilateral, as well as peripheral, participation is seen. These characteristics, according to [81,82], are primarily associated with bacterial pneumonia.
- According to [9,85], COVID-19 and typical viral pneumonia have many similarities, including bilateral GGOs and patchy consolidations. Peripheral and diffuse distributions, vascular thickening, micro-reticular opacity, and typical viral-like Ground Glass Opacities (GGOs) are some of the more likely hallmarks of COVID-19-induced pneumonia [86,87]. By closely inspecting the generated heatmap from several COVID-19 infected X-rays (Figure 13), it is observed that the opacities are distributed in a peripheral and diffuse manner. Furthermore, vascular thickening, as well as other conventional viral features, is localized in some of the cases.

Previous studies show that deep learning models have experienced better accuracy in the detection and diagnosis of COVID-19 positive and negative cases from the chest X-ray images in this current situation. Moreover, in the near future, the deep learning model will also play a vital role to combat the pandemic. A plan for future work is to explore federated learning on blockchain technology [88] to build a distributed trust-less COVID-19 detection system.

Despite the fact that we collected a huge number of chest X-ray images to train our model, it still has to be tested with a large number of patient images from various nations to ensure its reliability. We started with ResNet50V2 as a basis model and then assembled some extra layers. Again, ResNet50V2 is a deeper model in general, and the suggested architecture becomes deeper and more complex by adding additional layers to the existing blocks. Although deeper models is good at extracting features, they take a long time to train with a large dataset. Hence, we plan to develop a deep learning model by applying image processing techniques (e.g., enhancing, filtering, segmenting etc.) on collected X-ray image datasets that are modest in complexity, practicable, robust and more accurate in the future.

## 6. Conclusions

As the number of COVID-19 cases is increasing at a high rate, many countries lack the testing resources to perform the expected number of tests daily. So, in the current situation, alternative approaches are required to be adopted to have a rapid diagnosis of COVID-19 cases to prevent this pandemic. Therefore, here, a deep learning model based on ResNet50V2 architecture for the detection and classification of COVID-19 cases considering X-ray images of the chest has been investigated. The architecture is not only experimented with a balanced dataset but also considered with an updated dataset gathered from different publicly available sources. The models are able to work with binary class and multi-class classifications. A Grad-CAM-based discriminative visualization approach is also applied to make the proposed model more explainable and interpretable. Based on the experimental evaluations, the proposed model achieved a promising result on the prepared datasets. The proposed system will help the radiologist and clinicians for the early detection of the virus by providing timely assistance to patients and also reducing the rate of community transmission.

## Figures and Tables

**Table 1:** Related literature on X-ray for the purpose of COVID-19 identification.

Author (Month, Year)	Number of Cases and Image	Training Model	VisualizeUsing Gard CAM/Other	Accuracy (%)
Krishnamraju K [45]	1000 COVID-19 and 1000 normal	VGG16+ MobileNet	No	97
Mousavi Z [46]	939 healthy cases, 800 COVID-19 and 942 viral pneumonia	Developed LSTM network	No	90
Luz [47]	1000 COVID-19, 1000 normal and 1000 pneumonia	Efficient deep learning model	Yes	93.9
Al-Waisy [48]	400 COVID-19 and 400 normal	COVID-CheXNet	Yes	99.99
Aslan [59]	1341 normal, 219 COVID-19 and 1345 viral pneumonia	mAlexNet +BiLSTM	No	98.14, for first architecture and 98.70
Chen [49]	3 datasets total 513 COVID-19 and1984 non-COVID-19	VGG16	Yes	98
Wang [50]	266 COVID-19, 8,066 normal and 5,538 pneumonia	COVID-Net	Yes	93.30
Gupta [51]	361 COVID-19, 1345 pneumonia and 1341 normal	InstaCovNet-19	Yes	99.08 for 3 class and 99.53 for 2 class
Arsenovic [60]	434 COVID-19, 1100 normal and 1100 bacterial pneumonia	ResNetCOVID-19	No	94.10
Ammar [52]	150 COVID-19, 150 normal, and 150 pneumonia	MobileNetV2, ResNet50V2, ResNet152V2, Xception, VGG16 and DenseNet12	No	Highest accuracy 91.28 for MobileNetV2
Jain [53]	490 COVID-19, 1345 normal and 3632 pneumonia	Xception net, Inception net V3 and ResNeXt,	No	Highest accuracy 97.97 for Xception
Mohammadi R [54]	181 COVID-19 and 364 normal	pre-trained VGG16, InceptionResNetV2, MobileNet and VGG19	No	Highest accuracy 99.1 MobileNet
Chowdhury [61]	1341 normal, 219 COVID-19 and 1345 viral pneumonia	PDCOVIDNet	Yes	96.58
Turkoglu [55]	219 COVID-19, 1583 normal and 4290 pneumonia	COVIDetectioNet	No	99.18
Makris [62]	112 COVID-19, 112 normal and 112 pneumonia	9 well-known pre-trained CNN model	No	95 for the best two model (Vgg16 and Vgg19)
Ouchicha [56]	1341 normal, 219 COVID-19 and 1345 viral pneumonia	CVDNet	No	96.69
Civit-Masot [63]	132 COVID-19, 132 healthy and 132 pneumonia	VGG16	No	86.00
Mahmud [64]	1583 normal, 305 COVID-19, 1493 viral pneumonia, 2780 bacterial pneumonia	CovXNet	Yes	90.2 accuracy for four class
Khan [57]	1203 normal, 290 COVID-19 931 viral pneumonia, 660 bacterial pneumonia	CoroNet	No	Overall accuracy of 89.6
Ozturk [20]	125 COVID-19 cases, 500 no findings, 500 pneumonia cases	DarkCovid-Net	Yes	98.08 for two class and 87.02

**Table 2:** Definition of notations.

Notations	Definition
I	denotes as input matrix (e.g., image)
K	represents 2D filter by considering dimension m × n
F	output of 2D characteristic map
I*K	operation of convolution
x	represents as input vector
Z	represents as output vector
Train_acc	represents as training accuracy
Val_acc	represents as validation accuracy
pneumonia_bac	represents bacterial pneumonia cases
pneumonia_vir	represents viral pneumonia cases

**Table 3:** Proposed model layer details.

Input Layer	Output Shape	Number of Trainable
	(Depth*Height*Weight)	Parameters
Input image	(3*224*224)	0
Conv1	(64*112*112)	9472
Maxpool1	(64*56*56)	0
Conv2	(256*28*28)	16,640
Maxpool2	(256*28*28)	0
Conv3	(512*14*14)	66,048
Maxpool3	(512*14*14)	0
Conv4	(1024*7*7)	263,168
Maxpool4	(1024*7*7)	0
Conv5	(2048*7*7)	1,050,624
FC (Batch_normalization)	(2048*7*7)	8192
FC (ReLu)	(2048*7*7)	0
“Concatanated layers”	-	-
FC1 (Flatten)	(100,352)	0
FC2 (Dropout)	(100,352)	0
FC3 (Flatten)	(100,352)	0
FC4 (Dropout)	(100,352)	0
FC5 (ReLu)	(256)	25,690,368
FC6 (SoftMax)	(4)	1028

**Table 4:** Dataset-1.

Subset	COVID-19	Normal	Viral Pneumonia	Bacterial Pneumonia
Training Data	686	690	690	690
Test Data	457	460	460	460
Total	1143	1150	1150	1150

**Table 5:** Dataset-2.

Subset	COVID-19	Normal	Viral Pneumonia	Bacterial Pneumonia
Training Data	1143	1150	1150	1150
Test Data	457	460	460	460
Total	1600	1610	1610	1610

**Table 6:** Class-wise performance results.

Class	Task	Precision (%)	Recall (%)	F1-Score (%)	Accuracy (%)
Four	COVID-19	99.0	94.0	97.0	88.79
	Normal	94.0	91.0	92.0	
	Bacterial Pneumonia	80.0	94.0	87.0	
	Viral Pneumonia	83.0	77.0	80.0	
	COVID-19	1.00	96.0	98.0	
Three	Normal	95.0	99.0	97.0	97.22
	Bacterial Pneumonia	97.0	96.0	97.0	
	COVID-19	1.00	97.0	98.0	
Three	Normal	86.0	98.0	91.0	93.11
	Viral Pneumonia	95.0	85.0	90.0	
	COVID-19	99.0	95.0	97.0	
Three	Viral Pneumonia	87.0	97.0	91.0	92.10
	Bacterial Pneumonia	95.0	85.0	90.0	
Two	COVID-19	99.0	99.0	99.0	99.13
	Normal	99.0	99.0	99.0	
Two	COVID-19	1.00	97.0	98.0	98.47
	Bacterial Pneumonia	97.0	1.00	98.0	
Two	COVID-19	1.00	98.0	99.0	98.91
	Viral Pneumonia	98.0	1.00	99.0	

**Table 7:** Classification performance between Dataset-1 and Dataset-2.

Dataset	Class	Task	Precision (%)	Recall (%)	F1-Score (%)	Accuracy (%)
Dataset-1	Four	COVID-19	99.0	94.0	97.0	88.79
		Normal	94.0	91.0	92.0	
		B. Pneumonia	80.0	94.0	87.0	
		V. Pneumonia	83.0	77.0	80.0	
Dataset-2	Four	COVID-19	1.00	1.00	1.00	99.46
		Normal	1.00	1.00	1.00	
		B. Pneumonia	1.00	98.0	99.0	
		V. Pneumonia	98.0	1.00	99.0	

**Table 8:** Training and validation results for different classes.

Class		Dataset-01		
	Train_acc	Val_acc	Train_loss	Val_loss
Three (COVID vs. pneu_vir vs. normal)	0.9912	0.9311	0.0088	0.0689
Two (COVID vs. pneu_vir)	0.9935	0.9891	0.0109	0.0065
Two (COVID vs. pneu_bac)	0.9974	0.9847	0.0026	0.0153

**Table 9:** Performance of the proposed model on each fold.

Folds	Precision(%)	Recall(%)	F1-Score%)	Accuracy%)
Fold-1	89.25	88.75	88.5	88.80
Fold-2	91.5	91.75	91.75	91.64
Fold-3	88.75	88.75	88.5	88.59
Fold-4	92.25	91.75	91.75	91.73
Fold-5	88.5	88.25	87.75	88.06
Average	90.05	89.85	89.65	89.76

**Table 10:** Performance of the proposed model on each fold considering two-class COVID-19 vs. adult pneumonia.

Folds	Precision	Recall	F1-Score	Accuracy(%)
Fold-1	0.985	0.985	0.985	98.48
Fold-2	0.99	0.99	0.99	98.91
Fold-3	0.975	0.975	0.98	97.61
Fold-4	0.985	0.985	0.98	98.04
Fold-5	0.985	0.985	0.98	98.26
Average	0.984	0.984	0.983	98.26

**Table 11:** Performance results comparison among the suggested model and the other traditional pre-trained models.

Model	Class	Precision (%)	Recall (%)	F1-Score (%)	Accuracy (%)
VGG19	4	90	88.5	89	88.98
ResNet50	4	88	87.25	87.5	87.5
InceptionV3	4	87.5	87.25	87.5	87.39
VGG19	3	93.25	94	93	94.35
ResNet50	3	95	93.5	94	94.88
InceptionV3	3	93	92.25	92.75	93.27
VGG19	2	99	98.5	98	98.5
ResNet50	2	99	98	98	98.33
InceptionV3	2	0.98	97	98	97.67
Proposed model (on Dataset-1)	4	90.05	89.85	89.65	89.76
	3	0.97	89.85	0.9633	97.22
	2	0.99	89.85	0.99	99.13
Proposed model (on Dataset-2)	4	99.5	99.4	99.5	99.46
Proposed model (on Dataset-3)	2	98.4	98.4	98.3	98.26

**Table 12:** Performance results comparison among the suggested model and the other previous state-of-the-art works.

Author	Architecture	Number of Images	Class	Accuracy (%)
Khan et al. [57]	CoroNet	297 COVID-19, 330 bacterial pneumonia, 310 normal, 327 viral pneumonia images.	4	89.6
			3	95
			2	99
Mahmud et al. [64]	CovXNet	305 COVID-19, 305 bacterial pneumonia, 305 normal, 305 viral pneumonia images.	4	90.3
			3	89.6
			2	94.7
Ammar et al. [52]	6 pre-trained models	150 COVID-19, 150 normal, 150 pneumonia images.	3	91.28
Mousavi Z et al. [46]	Developed LSTM network	800 COVID-19, 942 viral pneumonia, 939 healthy cases images.	3	90.0
Arsenovic et al. [60]	ResNetCOVID-19	434 COVID-19, 1100 normal, 1100 bacterial pneumonia.	3	94.1
Hemdan et al. [79]	COVIDXNet	25 COVID-19 and 25 normal images.	2	90
Sethy et al. [58]	ResNet50 plus SVM	25 COVID-19 and 25 non-COVID-19.	2	95.38
Proposed model (Dataset-1)	Tuned ResNet50V2	1143 COVID-19, 1150 viral pneumonia, 1150 bacterial pneumonia, 1150 normal images.	4	89.76
			3	97.22
			2	99.13
Proposed model (Dataset-2)	Tuned ResNet50V2	1143 COVID-19, 1150 viral pneumonia, 1150 bacterial pneumonia, 1150 normal images.	4	99.46
Proposed model (Dataset-3)	Tuned ResNet50V2	1143 COVID-19, 1150 adult pneumonia.	2	98.26

## Data Availability

The datasets used in this investigation are available on request from the corresponding author.
